# Exertional Rhabdomyolysis after Military Training Paralleled by Systemic Microvascular Dysfunction and Plasma Cytokine Increase: A Case Report

**DOI:** 10.5935/abc.20190165

**Published:** 2019-08

**Authors:** Flavio Pereira, Roger de Moraes, Diogo Bavel, Andrea Rocha de Lorenzo, Eduardo Tibirica

**Affiliations:** 1 Instituto Nacional de Cardiologia, Rio de Janeiro, RJ - Brazil; 2 Universidade Estácio de Sá, Rio de Janeiro, RJ - Brazil

**Keywords:** Rhabdomyolosis, Exercise, High-Intensity Interval Training, Myalgia, Cytokinase/blood, Creatine Kinase, Muscle, Skeletal

## Introduction

Exertional rhabdomyolysis (ER) is diagnosed by the presence of intense muscular pain and sudden elevation of total plasma levels of the enzyme creatine kinase (CK), with or without myoglobinuria,^1^ is closely associated with acute fatigue during exercise,^2^ as well to the associated risk of acute renal injury, disseminated intravascular coagulation, cardiac arrhythmias, and electrolyte disturbances.^3^

ER is highly prevalent in military training, particularly when performed in adverse climatic conditions, and many cases progress rapidly to acute, life-threatening renal failure. Moreover, it is estimated that about one-third of the cases of ER involve young male afro-descendants with low physical conditioning and extreme dehydration, occurring during summer military training courses.^4^ In those situations, clinically healthy young subjects are submitted to strenuous exercise routines performed with combat uniforms and equipment and without adequate hydration possibilities.^4^

The assessment of systemic endothelial microvascular reactivity has already been proven to be essential in the investigation of the pathophysiology of cardiovascular and metabolic diseases.^5^ Additionally, the cutaneous microcirculation is now considered as an accessible and representative vascular bed for the assessment of systemic microcirculatory reactivity and density.^5^ Considering that ER has already been shown to be related to decreased systemic endothelium-dependent vasodilation in the systemic circulation in the experimental setting,^6^ it is reasonable to speculate that ER is also associated with significant systemic microcirculatory dysfunction. Moreover, there is no description in the specialized literature of the association of ER with microvascular endothelial function is humans. To the best of our knowledge, this is the first report on the detrimental outcomes of ER on endothelium-dependent systemic microvascular reactivity in human beings.

### Case report

This case report is part of an observational research study without any intervention investigating the impact of special military training courses on cytokine profile and microvascular reactivity and the risk of developing ER in Brazilian Air Force military personnel who fully completed a five-week training period. This study was performed in accordance with the Declaration of Helsinki of 1975 (revised in 2013). The case report was approved by the Institutional Review Board (IRB) of the National Institute of Cardiology of the Ministry of Health, Rio de Janeiro, Brazil under protocol number # CAAE 49792515.6.0000.5272. The subject read and signed the informed consent form approved by the IRB. The patient was encouraged to share his perception of the clinical event that occurred during special military training with his colleagues.

The patient was a 21-year-old and physically fit afro-descendant Brazilian Air Force military trainee, who spontaneously applied for riot control military training. The military was considered to have excellent aerobic endurance for his age range (20-29 years of age), using the Cooper run test (VO_2_max of 54.66 ml/kg/min). The patient presented no significant information on past personal or familial past medical history, including ER, and did not use any medication nor oral supplements during the period of military training. We tested the patient’s blood using hemoglobin electrophoresis, which showed the absence of hemoglobin S. Thus, we can consider that the patient did not present sickle-cell trait. He was diagnosed with ER on the second day of military training. He had performed running exercise in a combat uniform and transporting a 15 Kg kit including shield and gun, with a limited intake of water, and after being exposed to tear and pepper gases for 45 minutes. On the day before, he had run 2,400 meters in 12 minutes, and on both occasions, the running exercises were performed in warm (32ºC) and humid (86% relative humidity) conditions typical of the summer season in Rio de Janeiro, Brazil.

The patient had vomiting, postural hypotension, myalgia and muscle weakness in the hip region and lower limbs and was promptly referred to the Air Force Hospital. He soon developed fever (41ºC axillary temperature), dark-colored urine, lower limb edema and gait difficulty.

The evaluations of microvascular reactivity were performed one day before the beginning of military training and one day after hospital discharge, both in the morning between 8 and 12 AM and after a 12-hour fast. Microcirculatory tests were performed after a 20-minute rest in the supine position in a temperature-controlled room (23 ± 1°C). Microvascular reactivity was evaluated using a laser speckle contrast imaging system (PeriCam PSI system, Perimed, Järfälla, Sweden) in combination with skin iontophoresis of acetylcholine (ACh) for noninvasive and continuous measurement of cutaneous microvascular perfusion changes (in arbitrary perfusion units, APU).^7^ During the post-occlusive reactive hyperemia (PORH) test, arterial occlusion was performed with supra-systolic pressure using a sphygmomanometer for 3 min. Following the release of pressure, the maximum flux was measured. Measurements of skin blood flow were divided by the mean arterial pressure to yield the cutaneous vascular conductance (CVC) in APU/mmHg. The capillary density, defined as the number of perfused capillaries per mm^2^ of skin area, was assessed by high-resolution intra-vital color microscopy (Moritex, Cambridge, UK). The dorsum of the non-dominant middle phalanx was used for image acquisition. Images were acquired and saved for posterior off-line analysis using a semi-automatic integrated system (Microvision Instruments, Evry, France). The mean capillary density was calculated as the arithmetic mean of the number of visible (i.e., spontaneously perfused) capillaries in three contiguous microscopic fields of 1 mm^2^ each, as described previously.^8^

Laboratory testing of the subject is shown in table 1. The plasma levels of the enzyme CK were more than 5 times higher than reference laboratory ranges, and together with the symptoms suggested the diagnosis of ER. The creatinine clearance, calculated using the Cockcroft-Gault formula, was markedly reduced. Treatment consisted primarily of intravenous infusion of saline solution (≥ 2,5 L/day) with bicarbonate for pH normalization and myoglobin washout^9^ and to maintain adequate urine output. The plasma cytokine analysis is presented in table 2, showing increased levels of IL-1b, IL-6, IL-10, IL-1Ra even after hospital discharge.

**Table 1:** Laboratory testing of the patient before military training, during hospitalization (D, days) and one day after hospital discharge

Parameters	BEFORE	D1	D2	D3	D4	D5	AFTER	Reference ranges
Red blood cells (10^6^/µL)	5.3	5.8	5.0	4.6	4.4	4.9	5.3	4.5 – 6.2
Hemoglobin (g/dL)	15.8	16.9	14.5	13.5	13.0	15.1	15.4	13.5 – 18.0
Hematocrit (%)	47.5	51.5	43.3	40.5	38.1	43.2	47.0	40 – 54
White Blood Cell Count (µL)	9,000	18,600	9,580	7,120	6,470	8,920	9,200	5,000 – 10,000
Platelet counts (x1000/µL)	278	313	233	201	195	267	269	150 – 450
Urea (mg/dL)	39	67	47	31	25	27	40	15 – 40
Creatinine (mg/dL)	1.05	2.1	1.3	1.6	1.4	1.2	1.03	0.6 – 1.2
Creatinine Clearance (mL/min)	121	60	98	80	106	123	120	97 – 137
Calcium (mmol/L)	2.34	10.9	9.8	8.7	8.2	9.6	2.72	2.23 – 2.55
Magnesium (mg/dL)	1.9	2.3	2.1	1.8	2.5	2.4	2.1	1.6 – 2.6
Sodium (mmol/L)	138	140	136	142	155	141	138	137 – 145
Potassium (mmol/L)	3.6	4.2	3.6	3.7	3.3	4.2	4.1	3.6 – 5.0
Creatine kinase (U/L)	370	1,100	2,116	1,496	306	211	158	30 – 170
TSH (µIU/mL)	2.10	-	-	-	-	-	2.60	0.35 – 4.94
T3 (ng/ml)	1.13	-	-	-	-	-	1.49	0.59 – 1.49
T4 (ng/dl)	1.19	-	-	-	-	-	1.16	0.70 – 1.48

T3: triiodothyronine; T4: thyroxine; TSH: Thyroid-Stimulating Hormone.

**Table 2:** Cytokine plasma levels (in pg/mL) of the patient before military training and one day after hospital discharge

Cytokines		BEFORE		AFTER
IL-1β		0.29		29.44
IL-6		0.41		0.74
IL-10		0.038		6.089
IL-1Ra		5.86		156.57
TNF-α		19.28		3.75
INF-γ		0.88		0.69

IL-1β: Interleukin-1 beta; IL-6: Interleukin-6; IL-10: Interleukin-10; IL-1Ra: IL-1 receptor antagonist; TNFα: tumour necrosis factor alpha; INF-γ: Interferon gamma.

**Figure 1:**
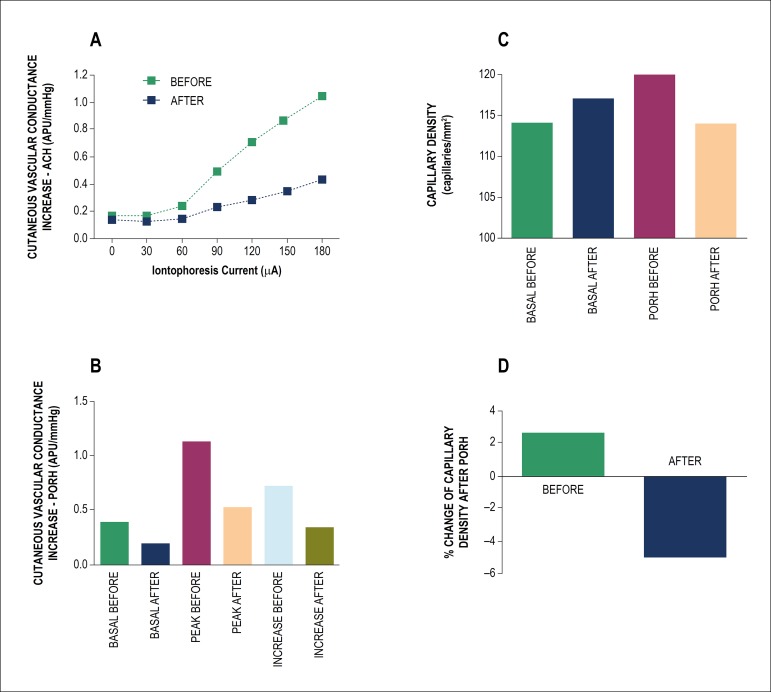
Effects of skin iontophoresis of acetylcholine (ACH) on cutaneous microvascular conductance (A, expressed in arbitrary perfusion units, APU, divided by mean arterial pressure, in mmHg) before military training (BEFORE) and one day after hospital discharge (AFTER). (B) Effects of forearm post-occlusive reactive hyperemia (PORH) on cutaneous microvascular conductance. (C) Functional capillary density before (BASAL) and during post-occlusive reactive hyperemia (PORH) before military training (BEFORE) and one day after hospital discharge (AFTER). (D) Percentage change in endothelial-dependent increase in capillary density after PORH before military training (BEFORE) and one day after hospital discharge (AFTER). BASAL: values before PORH; PEAK: maximum values after PORH; INCREASE: the difference between peak and basal values.

Of note, one day after hospital discharge, systemic endothelium-dependent microvascular reactivity was severely impaired. These results can be observed both in the pharmacological (acetylcholine-induced) and physiological (PORH-induced) microvascular vasodilator responses (Figure 1). Finally, cutaneous endothelium-dependent capillary recruitment was also impaired (Figure 1).

After six days of hospitalization and two additional weeks of rest at home, the subject had fully recovered and was able to return to his normal activities. Prognosis of ER is generally good if full recovery occurs.^9^

## Discussion

This case report demonstrates that ER after strenuous physical exercise, performed in adverse environmental conditions and with limited water access, can be paralleled by persistent systemic microvascular dysfunction, detectable up to 1 week after the beginning of symptoms and even after normalization of muscle enzymes and complete resolution of renal dysfunction. In fact, a marked reduction of the endothelium-dependent systemic microvascular reactivity induced by both pharmacological (acetylcholine) and physiological (post-occlusive reactive hyperemia, PORH) stimuli was observed one week after the diagnosis of ER. Moreover, skin capillary function, measured as post-ischemic capillary recruitment, was also significantly impaired, suggesting a loss of vasodilatory reserve and autoregulatory capacity and the existence of severe microvascular endothelial dysfunction.

Exercise training of moderate intensity is well-known to induce beneficial effects on the occurrence of cardiovascular diseases through the preservation of vascular endothelial function.^10^ On the other hand, strenuous exercise increases oxidative metabolism and produces a pro-oxidant environment, and consequent endothelial dysfunction,^11^ while regular and moderate physical activity promotes an antioxidant state and preserves endothelial function.^10^ Thus, high-intensity exercise training in previously untrained individuals, such as that of special military training, could be detrimental to the promotion of vascular health.

The microvascular alterations described above were simultaneous with alterations of the profile of plasma cytokines. Nevertheless, it is clearly not possible to establish a link between both phenomena in the present case report, since other metabolic changes could also be involved in the initiation of microvascular dysfunction. It is well known that ER is acutely associated with the production of pro-inflammatory cytokines.^12^ Even though we did not obtain cytokine plasma levels on the day of hospital admission, we observed an increase both in pro-inflammatory (IL-1b, IL-6) and anti-inflammatory (IL-10, IL-1Ra) cytokines after hospital discharge, compared to values obtained immediately before military training. The plasma levels of muscle-derived IL-6, which is considered to be a key mediator released during exhaustive exercise,^13^ usually starts to increase within the first hour of prolonged exercise and continues to rise depending on the duration of the exercise.^13^ In fact, it is well established that the elevation of pro-inflammatory cytokines at the time of muscle injury influences the synthesis of acute phase proteins and the expression of anti-inflammatory cytokines, as a physiological response to offset the inflammatory response.^13^ Moreover, it has been consistently shown that there is a rise in anti-inflammatory cytokines, IL-1ra and IL-10, following endurance exercise lasting longer than 2 h.^13^ Yet, the plasma levels of TNF-a were not increased one week after ER, suggesting that this cytokine has a different kinetics profile, compared with the aforementioned pro-inflammatory cytokines. Plasma levels of INF-g did not show important variations in the present case. Actually, most studies in the literature failed to demonstrate a significant rise in plasma IFN-g after exercise.^13^

Strengths and limitations of our experimental approach should be considered. The use of laser-based skin microvascular flowmetry, as well as the evaluation of the levels of plasma cytokines, is not yet possible in clinical practice. One major strength of the present case report is the demonstration of persistent systemic endothelial microvascular dysfunction and systemic inflammatory reaction after clinical and laboratory regression of ER. The long-lasting vascular inflammatory process observed in the present clinical case could have implications in the prognosis of patients presenting with ER. Nevertheless, it was impossible to retest these parameters in longer time intervals in the present case.

## Conclusion

ER may be accompanied by systemic microvascular dysfunction even after the resolution of symptoms and normalization of conventional laboratory tests. The microcirculatory disturbance is concurrent with alterations of plasma levels of both pro- and anti-inflammatory cytokines. Accordingly, ER should always be considered in the clinical scenario of muscle pain and disability, fever and dark urine after heavy exercise, including that performed for professional reasons. Besides that, the case report shows that ER may be associated with other complex and potentially severe conditions, which are microvascular dysfunction and systemic inflammation. These are novel findings which we would like to add to the clinicians´ reasoning. If the evaluation of microvascular function is made available clinically, it may be another potentially interesting evaluation to be performed in patients with ER. Nevertheless, more studies are needed to clarify the association between microvascular dysfunction and ER, as well as its clinical implications.
